# ﻿Seven new karyotypes for subfamily Cercosaurinae (Squamata, Gymnophthalmidae) with a synthesis of chromosomal data

**DOI:** 10.3897/compcytogen.20.170539

**Published:** 2026-01-09

**Authors:** Lucas S. F. Rachid, Yatiyo Yonenaga-Yassuda, Miguel Trefaut Rodrigues, Katia Cristina Machado Pellegrino

**Affiliations:** 1 Departamento de Ecologia e Biologia Evolutiva, Universidade Federal de São Paulo, Campus Diadema, Avenida Professor Artur Riedel, 275, Jardim Eldorado, CEP 09972-270, Diadema, SP, Brazil; 2 Departamento de Zoologia, Instituto de Biociências, Universidade de São Paulo, CEP 05508-090, São Paulo, SP, Brazil; 3 Departamento de Genética e Biologia Evolutiva, Instituto de Biociências, Universidade de São Paulo, CEP 05508-090, São Paulo, SP, Brazil

**Keywords:** Amazonia, Atlantic Forest, Neotropical, chromosomal evolution, chromosomal rearrangements, differential staining, Gymnophthalmoidea, phylogeny, taxonomy

## Abstract

Gymnophthalmidae is a monophyletic family of currently 297 Neotropical lizard species. Over the past 25 years, molecular studies have redefined previous morphology-based classifications, confirming the family’s monophyly and revealing three major clades plus Rachisaurinae: ((Riolaminae(Rachisaurinae(Gymnophthalminae)))(Cercosaurinae)). Despite increased taxonomic efforts, especially in the tribes Bachiini and Cercosaurini, of Cercosaurinae, cytogenetic data remain limited. Of over 200 species in these tribes, only three had published karyotypes. Here, we provide new karyotypic data for seven species of Cercosaurini using standard cytogenetic techniques (Ag-NOR, C- and RBG-banding). Diploid numbers ranged from 2n = 32 in *Bachia dorbignyi* (Duméril et Bibron, 1839) to 2n = 58 in *Placosoma glabellum* (Peters, 1870). Almost all species displayed karyotypes composed of macro- and microchromosomes, that varied in morphology. Ag-NORs were observed on macro- or microchromosomes in several species, with notable variability in *Bachia bresslaui* (Amaral, 1935). Constitutive heterochromatin was mostly restricted to centromeric or telomeric regions. In *Bachia dorbignyi*, we found a putative XX:XY system due to the presence of a dot-like microchromosome exclusively in male specimens. These results comprise the threefold amount of cytogenetic data available for Bachiini and Cercosaurini and help fill a major gap into our understanding of diversity and chromosome evolution within Gymnophthalmidae.

## ﻿Introduction

The Family Gymnophthalmidae Fitzinger, 1826, commonly known as “microteiids,” comprises lizards endemic to the Neotropical region, where they typically inhabit the leaf litter of subtropical and tropical forests in northern South America (Recoder et al. 2020; Marques-Souza et al. 2022; Vásquez-Restrepo et al. 2024a, b). This family reunites currently 297 species distributed across 56 genera (Uetz et al. 2025), and most attempts at grouping Gymnophthalmidae species based solely on morphological data (e.g. Ruibal 1952; Uzzell 1966) have been significantly revised over the past 25 years. Pellegrino et al. (2001) proposed the first molecular-based systematic arrangement for the group, using mitochondrial and nuclear sequences, and confirmed the monophyly of the family. This scheme was largely followed (e.g. Goicoechea et al. 2016; Recoder et al. 2020) until recent molecular phylogenies based on a larger taxonomic sampling have indicated that the genus *Alopoglossus* Boulenger, 1885 – now assigned to the family Alopoglossidae (Goicoechea et al. 2016; Hernández-Morales et al. 2020) – was recovered as the sister group to all other microteiids. The Gymnophthalmidae species remained allocated into four main clades: (I) subfamily Gymnophthalminae, tribes Heterodactylini, Iphisini and Gymnophthalmini; (II) Cercosaurinae, tribes Cercosaurini, Bachiini and Ecpleopodini; (III) Rachisaurinae, and (IV) Riolaminae (Pellegrino et al. 2001; Colli et al. 2015; Recoder et al. 2020). Molecular phylogenetic hypotheses suggested that several morphological traits evolved independently across different clades within the family, such as loss of the external ear, body elongation, and limb reduction. However, eyelid loss was restricted to Gymnophthalmini and does not represent a case of convergent evolution (Pellegrino et al. 2001).

In the last ten years, integrative taxonomic efforts focused on the tribes Bachiini Colli, Hoogmoed, Cannatella, Cassimiro, Gomes, Ghellere, Nunes, Pellegrino, Salerno, Souza et Rodrigues, 2015 and Cercosaurini Gray, 1838, which together comprise approximately 204 species, have resulted in the description of ten new genera (*Andinosaura* Sánchez-Pacheco, Torres-Carvajal, Aguirre-Peñafiel, Nunes, Verrastro, Rivas, Rodrigues, Grant et Murphy, 2017; *Centrosaura* Vásquez-Restrepo, Ibáñez, Sánchez-Pacheco et Daza, 2019; *Dendrosauridion* Lehr, Moravec, Lundberg, Köhler, Catenazzi et Šmíd, 2019; *Gelanesaurus* Torres-Carvajal, Lobos, Venegas, Chávez, Aguirre-Peñafiel, Zurita et Echevarría, 2016; *Kataphraktosaurus* Rojas-Runjaic, Barrio-Amorós, Señaris, Riva et Castroviejo-Fisher, 2021; *Magdalenasaura* Fang, Vásquez-Restrepo et Daza, 2020; *Potamites* Doan et Castoe, 2005; *Rheosaurus* Vásquez-Restrepo, Ibáñez, Sánchez-Pacheco et Daza, 2019; *Selvasaura* Moravec, Šmíd, Štundl, et Lehr, 2018 and *Wilsonosaura* Lehr, Moravec et von May, 2020) and around 46 new species. Other studies suggest that some clades may comprise species complexes, emphasizing the considerable number of taxa that are still awaiting formal description. In the genus *Bachia* Gray, 1838 and *Cercosaura* Wagler, 1830, two recognized species complexes – currently treated as subspecies (*Bachia monodactylus monodactylus* Dixon, 1973 and *B. monodactylus parkeri* Dixon, 1973; Murphy et al. 2019); *Cercosaura schreibersii albostrigatus* (Griffin, 1917) and *C. schreibersii schreibersii* Wiegmann, 1834 (Torres-Carvajal et al. 2016) – have not yet been evaluated through an integrative taxonomic approach. This underscores the urgent need for further taxonomic and systematic studies to better assess the diversity and evolutionary history of the group, particularly in the context of species conservation.

Despite their underscored diversity, only 26 species of microteiids have been karyotyped to date (less than 10% of the known species), largely due to their extremely small body size, rarity, and difficulties associated with field collection. Pioneering cytogenetic studies employing differential staining techniques such as Ag-NOR (nucleolus organizer regions), C-banding (CBG), and R-banding (RGB) in gymnophthalmids began in the mid-1990s and early 2000s (Table 1). These initial investigations were primarily conducted on species belonging to the tribes Gymnophthalmini and Ecpleopodini by Yonenaga-Yassuda and her research group (Pellegrino et al. 1999a, b, 2003; Yonenaga-Yassuda et al. 1995, 1996, 2005; Yonenaga-Yassuda and Rodrigues 1999; Laguna et al. 2010; Table 1). These studies revealed an enormous variation in the diploid number within Gymnophthalmidae, ranging from 2n = 34 in *Gymnophthalmus pleii* Bocourt, 1881 to 2n = 62 in *Nothobachia ablephara* Rodrigues, 1984 (Cole et al. 1990; Pellegrino et al. 1999a, respectively). Further, these works also showed two distinct karyotype patterns in the family: one characterized by a clear size distinction between macro- and microchromosomes, and another consisting of high diploid numbers with chromosomes decreasing gradually in size (e.g. Pellegrino et al. 1999a, b). Regarding sex chromosomes, two forms of male heterogamety have been documented in microteiids: a standard XX:XY system (e.g. *Nothobachia*, Pellegrino et al. 1999a) and X₁X₁X₂X₂:X₁X₂Y multiple sex chromosome system (e.g. *Calyptommatus* Rodrigues, 1991; Yonenaga-Yassuda et al. 2005). Furthermore, supernumerary chromosomes (Bs) have also been described in the group (e.g. *Micrablepharus* Dunn, 1932; Yonenaga-Yassuda and Rodrigues 1999 and *Nothobachia* Rodrigues, 1984; Pellegrino et al. 1999a). Lastly, diploid and triploid parthenogenetic lineages have also been identified in *Gymnophthalmus* Merrem, 1820 (2n; Cole et al. 1990) and *Loxopholis* Cope, 1869 (2n and 3n; Pellegrino et al. 2003, 2011; Laguna et al. 2010; Brunes et al. 2019). Such high variability of karyotypes makes gymnophthalmid lizards an intriguing group to investigate chromosomal evolution and broader evolutionary biology questions, as the role of hybridization in species diversification and parthenogenesis (Brunes et al. 2019).

**Table 1.** Karyotype data compiled from the literature for the Gymnophthalmidae. Subfamily, Tribe, Genus, Species, Species (initial description), diploid number (2n), macrochromosome (M) and microchromosomes (m), chromosomes with decreasing size (CDS), nucleolus organizer region (NOR), reference. For data not available in the literature, “x” is indicated. NA = not applicable.

Subfamily	Tribe	Genus	Species	Species (initial description)	2n	NOR	Reference
Cercosaurinae	Bachiini	* Bachia *	*Bachia bresslaui*	NA	2n = 46 (18M + 28m)	On macros/micros	Present work
			*Bachia dorbignyi*	NA	2n = 32 (18M + 14m; putative XX:XY)	x	Present work
	Cercosaurini	* Anadia *	*Anadia bitaeniata*	*Anadia bitaeniata*	2n = 46 (20M + 26m)	x	Gorman, 1970
		* Cercosaura *	*Cercosaura olivacea*	NA	2n = 42 (18M + 24m)	On micros	Present work
			*Cercosaura schreibersii albostrigatus*	NA	2n = 44 (20M + 24m)	x	Present work
		* Neusticurus *	*Neusticurus bicarinatus*	NA	2n = 44 (20M + 24m)	One macrochromosome pair	Present work
		* Placosoma *	*Placosoma cordylinum*	NA	2n = 44 (20M + 24m)	x	Present work
			*Placosoma glabellum*	NA	2n = 58 CDS	One macrochromosome pair	Present work
		* Potamites *	*Potamites ecpleopus*	*Neusticurus ecpleopus*	2n = 44 (20M + 24m)	x	Sherbrooke and Cole 1972
			*Potamites trachodus*	*Neusticurus strangulatus trachodus*	2n = 44 (20M + 24m)	x	Sherbrooke and Cole 1972
	Ecpleopodini	* Anotosaura *	*Anotosaura collaris*	*Anotosaura collaris*	2n = 44 (20M + 24m)	x	Rodrigues et al. 2013
		* Leposoma *	*Leposoma scincoides*	*Leposoma scincoides*	2n = 52 CDS	Pair 19	Pellegrino et al. 1999b
		* Loxopholis *	*Loxopholis ferreirai*	*Leposoma ferreirai*	2n = 44 (20M + 24m)	One microchromosome pair	Laguna et al. 2010
			*Loxopholis guianense*	*Leposoma guianense*	2n = 44 (20M + 24m)	One microchromosome pair	Pellegrino et al. 1999b
			*Loxopholis osvaldoi*	*Leposoma osvaldoi*	2n = 44 (20M + 24m)	One macrochromosome pair	Pellegrino et al. 1999b
			*Loxopholis percarinatum*	*Leposoma percarinatum*	2n = 44 (20M + 24m), 3n = 66 (30M + 36m)	One microchromosome pair (diploid form)	Pellegrino et al. 2003; Laguna et al. 2010
Gymnophthalminae	Gymnophthalmini	* Calyptommatus *	*Calyptommatus leiolepis*	*Calyptommatus leiolepis*	♂: 2n = 57 \ ♀: 2n = 58 (X_1_X_1_Y:X_1_X_1_X_2_X_2_)	Pair 27	Yonenaga-Yassuda et al. 2005
			*Calyptommatus nicterus*	*Calyptommatus nicterus*	♂: 2n = 57 \ ♀: 2n = 58 (X_1_X_1_Y:X_1_X_1_X_2_X_2_)	Pair 27	Yonenaga-Yassuda et al. 2005
			*Calyptommatus sinebrachiatus*	*Calyptommatus sinebrachiatus*	♂: 2n = 57	x	Yonenaga-Yassuda et al. 2005
Gymnophthalminae	Gymnophthalmini	* Gymnophthalmus *	*Gymnophthalmus cryptus*	*Gymnophthalmus cryptus*	2n = 44 (20M + 24m)	x	Cole et al. 1993
			*Gymnophthalmus leucomystax*	*Gymnophthalmus leucomystax*	2n = 44 (20M + 24m)	On micros	Yonenaga-Yassuda et al. 1995
			*Gymnophthalmus pleii*	*Gymnophthalmus pleii*	2n = 34 (12M + 22m; XX:XY)	x	Cole et al. 1990
			*Gymnophthalmus speciosus*	*Gymnophthalmus speciosus*	2n = 44 (20M + 24m)	x	Cole et al. 1990
			*Gymnophthalmus underwoodi*	*Gymnophthalmus underwoodi*	2n = 44 (20M + 24m)	One medium size chromosome	Cole et al. 1990; Yonenaga-Yassuda et al. 1995
			*Gymnophthalmus vanzoi*	*Gymnophthalmus* sp.	2n = 44 (20M + 24m)	On micros	Yonenaga-Yassuda et al. 1995
		* Micrablepharus *	*Micrablepharus atticolus*	*Micrablepharus atticolus*	2n = 50, 51, 52, 53 CDS (1B, 2B, 3B)	On macros/micros	Yonenaga-Yassuda and Rodrigues 1999
			*Micrablepharus maximiliani*	*Micrablepharus maximiliani*	2n = 50, 51 CDS (1B, 2B)	On macros/micros	Yonenaga-Yassuda and Rodrigues 1999
		* Nothobachia *	*Nothobachia ablephara*	*Nothobachia ablephara*	2n = 62, 63, 64 CDS (1B, 2B)	On macros/micros	Pellegrino et al. 1999a
		* Procellosaurinus *	*Procellosaurinus erythrocercus*	*Procellosaurinus erythrocercus*	2n = 40 (16M + 24m)	One microchromosome pair	Yonenaga-Yassuda et al. 1996
			*Procellosaurinus tetradactylus*	*Procellosaurinus tetradactylus*	2n = 40 (16M + 24m)	One microchromosome pair	Yonenaga-Yassuda et al. 1996
		* Psilops *	*Psilops paeminosus*	*Psilophthalmus paeminosus*	2n = 44 (20M + 24m)	One microchromosome pair	Yonenaga-Yassuda et al. 2005
		* Tretioscincus *	*Tretioscincus oriximinensis*	*Tretioscincus oriximinensis*	2n = 42 (18M + 24m)	One microchromosome pair	Yonenaga-Yassuda et al. 2005
		* Vanzosaura *	*Vanzosaura multiscutata*	*Vanzosaura rubricauda*	2n = 40 (16M + 24m)	On macros/micros	Yonenaga-Yassuda et al. 1996

**Figure 1.**
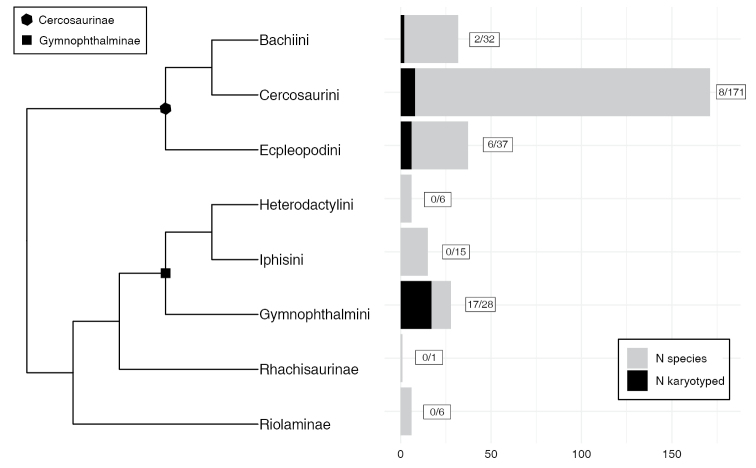
Number of Gymnophthalmidae species with available karyotypic data, including those generated in this work (see Table 1) shown by tribe and subfamily, relative to the total number of described species in each clade. Phylogenetic topology adapted from Recoder et al. (2020).

The disproportionately low number of species of gymnophthalmids with known karyotypes (Fig. 1; Table 1), in comparison to their recognized taxonomic diversity, emphasizes a considerable gap in our understanding of chromosomal evolution within the family. Up to now, no chromosomal data has been reported for any species of the genus *Bachia*. Within the tribe Cercosaurini, such data are available for only three species: *Anadia bitaeniata* Boulenger, 1903 (2n = 46; 20M + 26m), *Potamites ecpleopus* (Cope, 1875), and *P. trachodus* (Uzzell, 1966), the latter two exhibiting a diploid number of 2n = 44 (20M + 24m) (Gorman 1970; Sherbrooke and Cole 1972, respectively). Therefore, among the 203 recognized species in the tribes Bachiini and Cercosaurini, only these three have published karyotypic information (Fig. 1). Furthermore, chromosomal banding patterns remain unreported for both tribes. The most recent cytogenetic study focusing on gymnophthalmids dates to the early 2010s, with chromosomal data reported for *Loxopholis percarinatum* (Müller, 1923) (Laguna et al. 2010), highlighting the slow pace of research in this field. To fill this knowledge gap, we report seven novel karyotypes from species of the tribes Cercosaurini and Bachiini, characterized by using conventional cytogenetic methods such as NOR staining, C-banding, and RBG-banding. Additionally, we provide a comprehensive compilation of chromosomal data for the family Gymnophthalmidae and discuss the current state of knowledge in this field, pointing up the critical role of chromosomal studies in elucidating the evolutionary history of this diverse and understudied lizard group.

## ﻿Material and methods

### ﻿Sampling

**Figure 2.**
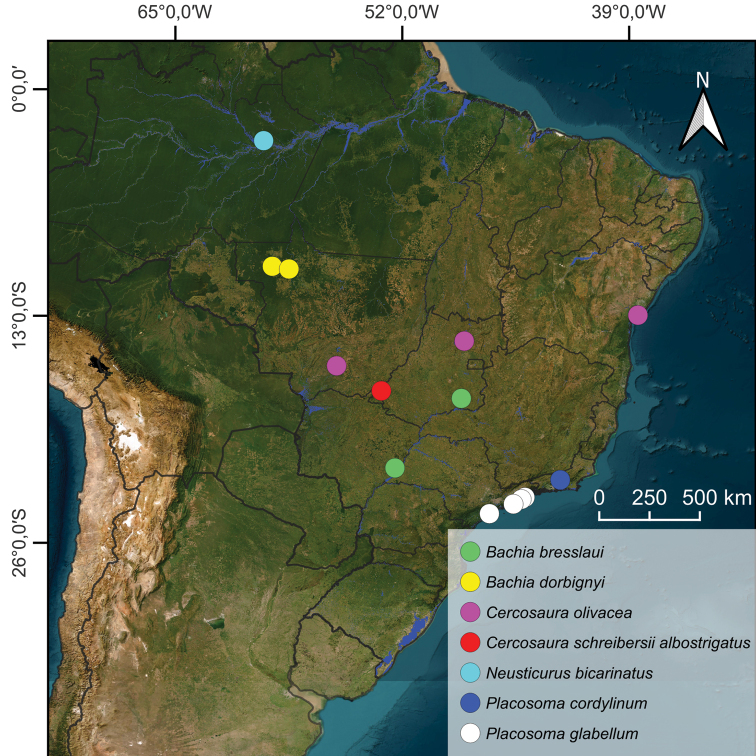
Localities of Cercosaurinae species sampled in this study. Map courtesy: Guilherme Honório Fernández (UFSCar).

**Figure 3.**
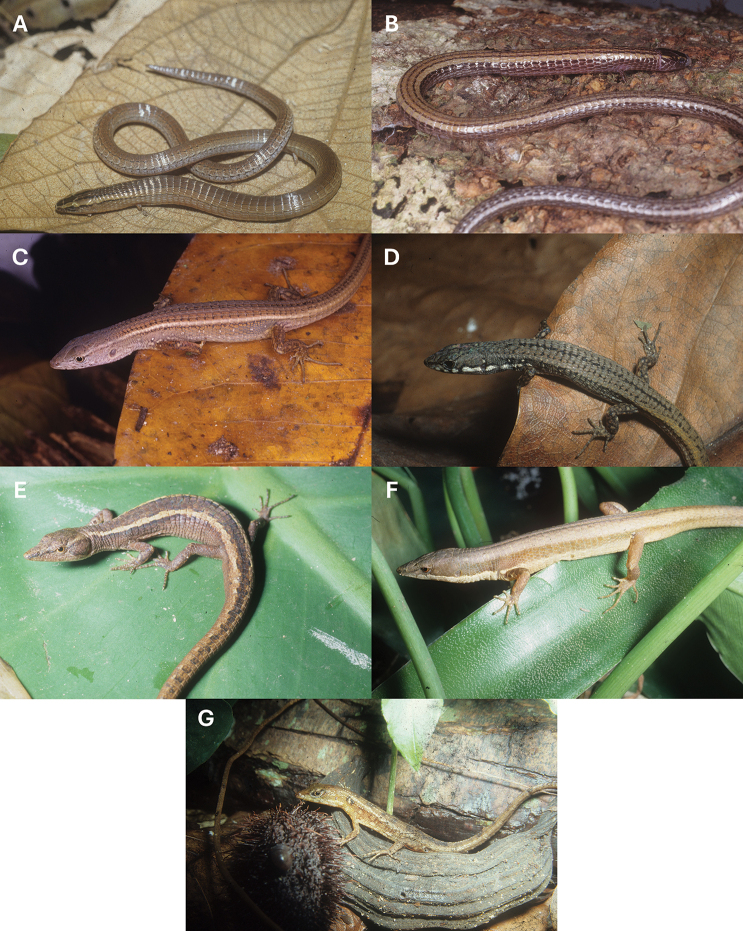
**A.** Specimen of *Bachia bresslaui* from Caldas Novas, Goiás, Brazil; **B.***Bachia dorbignyi* from Aripuanã, Mato Grosso, Brazil; **C.***Cercosaura olivacea* from Serra da Mesa, Goiás, Brazil; **D.***Cercosaura schreibersii albostrigatus* from Alto Araguaia, Mato Grosso, Brazil; **E.***Placosoma cordylinum* from Teresópolis, Rio de Janeiro, Brazil; **F.***Placosoma glabellum* from Ubatuba, São Paulo, Brazil; **G.***Neusticurus bicarinatus* from Manaus, Amazonas, Brazil. Photos not on scale. Photographs by MTR.

The new karyotypes obtained correspond to one female and one male of *Bachia bresslaui* (Amaral, 1935), one female and two males of *Bachia dorbignyi* (Duméril et Bibron, 1839), one female and four males of *Cercosaura olivacea* (Gray, 1845), one male *Cercosaura schreibersii albostrigatus* (Griffin, 1917), one male of *Neusticurus bicarinatus* (Linnaeus, 1758), three males of *Placosoma cordylinum* Tschudi, 1847 and three females and four males of *Placosoma glabellum* (Peters, 1870) (Figs 2, 3). The localities, field numbers, and vouchers of museums are listed in Suppl. material 1. The specimens were fixed in a 10% formalin, stored in 70% ethanol and deposited in the Museu de Zoologia da Universidade de São Paulo (MZUSP).

### ﻿Chromosome preparations and staining techniques

Chromosome preparations were obtained from intestine, testes, bone marrow, spleen, and liver according to routine techniques (e.g. Ford and Hamerton 1956; Schmid 1978), or from fibroblast cultures (Yonenaga-Yassuda et al. 1988). Chromosomes were analyzed after Giemsa staining, C-banding (Sumner 1972) and Ag-NOR staining (Howell and Black 1980). For replication R-banding, the cells were treated with 5-BrdU (final concentration 25 μg/ml) for 8–9 hours before harvesting, followed by FPG staining (Dutrillaux and Couturier 1981). The slides were analyzed under a Zeiss AxioPhot microscope.

### ﻿Ethical approval

The animals were collected under permits granted by the Instituto Chico Mendes de Conservação da Biodiversidade (ICMBio #10126) and this study was approved by the Ethics Committee for Animal Use of the Universidade Federal de São Paulo (UNIFESP) (#5237050922, ID #012264).

## ﻿Results

### ﻿Karyotypes description and Ag-NOR sites

***Bachia bresslaui* (2n = 46, 18M + 28m)**

**Figure 4.**
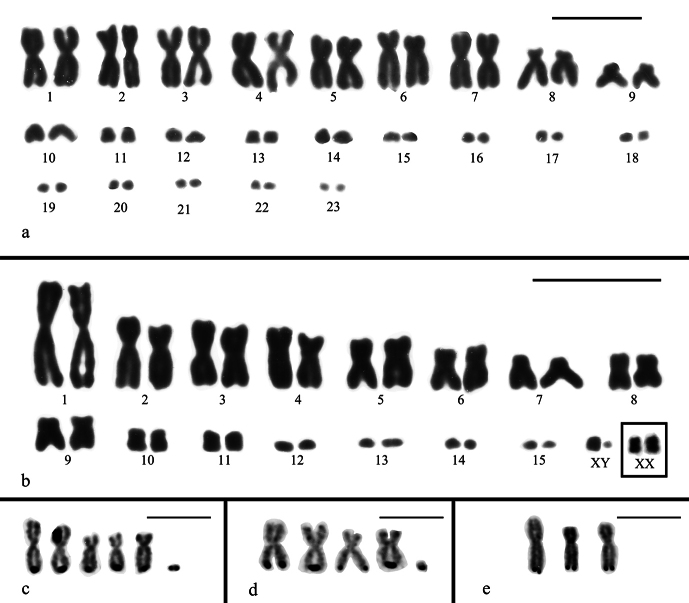
Giemsa-stained karyotypes of **a.***Bachia bresslaui* (2n = 46, 18M + 28m) and **b.***Bachia dorbignyi* (2n = 32, 18M + 14m; XX:XY) in inset the XX chromosome in female specimen. **c–e.**Ag-NOR stained chromosomes of *Bachia bresslaui*; **c.** 6 Ag-NORs at telomeric region of the long arm of five metacentric/submetacentric macrochromosomes and a single microchromosome; **d.** 5 Ag-NORs at telomeric region of the long arm of four metacentric/submetacentric macrochromosomes and one microchromosome; **e.** 3 Ag-NORs at telomeric region of the long arm of three metacentric/submetacentric macrochromosomes. Scale bar: 10 μm.

The analysis of 20 conventionally stained metaphases revealed a karyotype composed of 18 biarmed macrochromosomes being metacentrics or submetacentrics, except for two subtelocentric pairs (8 and 9). There are 14 pairs of microchromosomes, most acrocentrics, but with at least two biarmed pairs, observed in high quality metaphases (Fig. 4a). Pair 10 is also acrocentric but shows an intermediate size between macrochromosome pair 9 and microchromosome pairs 11–23. A total of 28 metaphases analyzed after Ag-staining revealed variability in number of NORs (from 2 to 6), which were located at telomeric regions of the long arm from one or both homologous of three pairs of macrochromosomes (Fig. 4c–e). In the female specimen, a single microchromosome also carried the Ag-NORs at least in 42% out of 19 metaphases analyzed. Heteromorphic sex chromosomes between the male and female specimens were not detected.

***Bachia dorbignyi* (2n = 32, 18M + 14m, putative XX:XY)**

In a total of 31 conventionally stained metaphases, we found a karyotype formed by 18 biarmed macrochromosomes including metacentrics, submetacentrics and subtelocentrics. The first two pairs of microchromosomes (pairs 10 and 11) are biarmed and larger than the remaining four acrocentric pairs (Fig. 4b). In both males, a dot-like microchromosome (probably a Y) was observed but it was absent in the female specimen. The X seems to be among the largest pairs of acrocentric microchromosomes after the pair 11 (Fig. 4b inset). Unfortunately, meiotic spreads from the male specimens were not available.

***Cercosaura olivacea* (2n = 42, 18M + 24m)**

**Figure 5.**
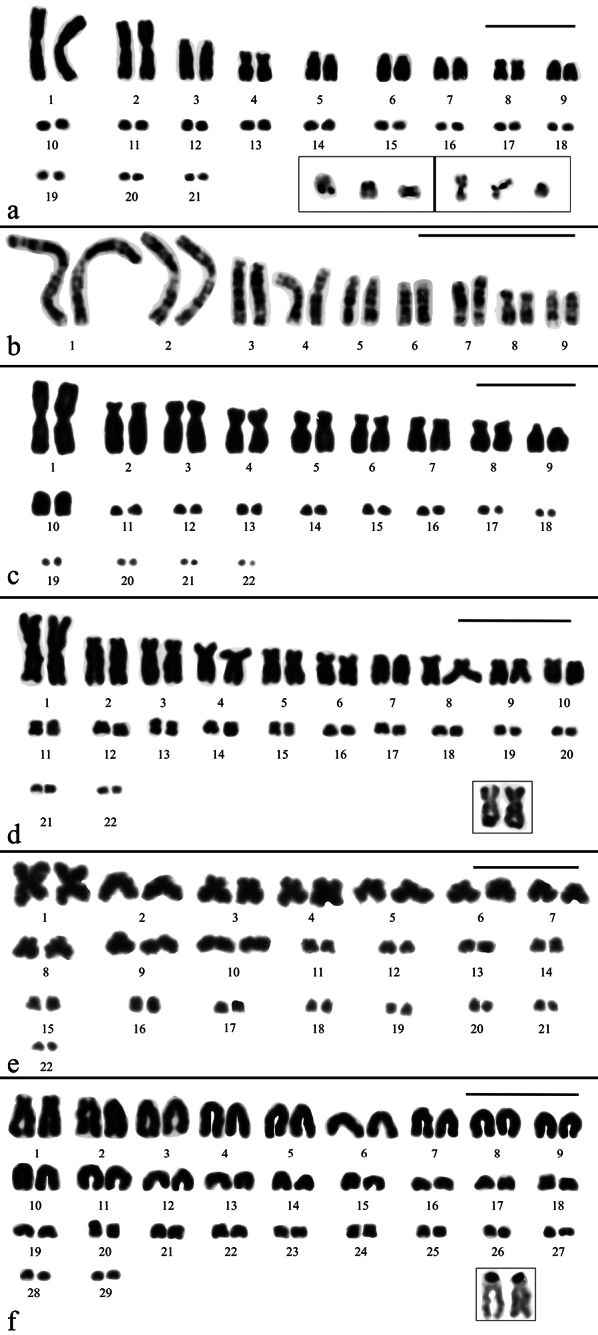
Karyotypes of Cercosaurini species after conventional and Ag-NOR staining. **a.***Cercosaura olivacea* (2n = 42, 18M + 24m) with inset showing 4 Ag-NORs (one pair in association) at telomeric region of microchromosome pairs (left) and 5 Ag-NORs (two pairs in association) at telomeric region of microchromosome pairs (right); **b.** RBG-banding of macrochromosomes pairs; **c.***Cercosaura schreibersii albostrigatus* (2n = 44, 20M + 24m); **d.***Neusticurus bicarinatus* (2n = 44, 20M + 24m). Inset the Ag-NOR at telomeres of long arm of a macrochromosome pair. **e.***Placosoma cordylinum* (2n = 44, 20M + 24m); **f.***Placosoma glabellum* (2n = 58) and Ag-NOR at telomeric region of short arm of a single macrochromosome pair (inset). Scale bar: 10 μm.

**Figure 6.**
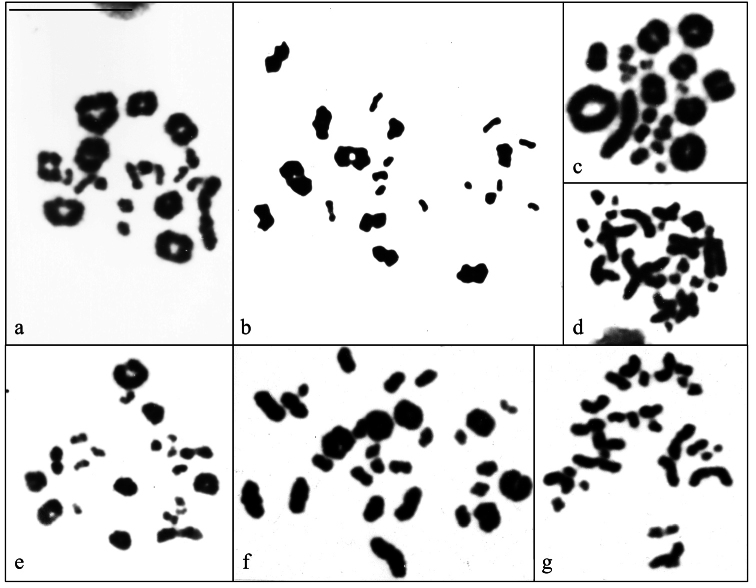
Diakinesis cells conventionally stained of **a.***Cercosaura olivacea* (21 bivalents: 9M + 12m); **b.***Cercosaura schreibersii albostrigatus* (22 bivalents: 10M + 12m); **c.***Neusticurus bicarinatus* (22 bivalents: 10M + 12m); **d.** Metaphase II of *N. bicarinatus* (22 chromosomes); **e.** Diakinesis of *Placosoma cordylinum* (22 bivalents: 10M + 12m); **f.** Diakinesis of *P. glabellum* (29 chromosomes) and **g.** Metaphase II of *P. glabellum* (29 chromosomes). Scale bar: 10 μm.

The analyses of 60 conventionally stained metaphases revealed a karyotype consisting of 42 chromosomes with 18 macrochromosomes and 24 microchromosomes (Fig. 5a). Among the macrochromosomes, pairs 1 and 2 are metacentrics, pairs 4 and 8 submetacentrics and pairs 3, 5 to 7 and 9 are acrocentrics. The 24 microchromosomes are acrocentrics. In a total of 49 Ag-stained metaphases, 4 (31 cells) or 5 (18 cells) small Ag-NOR were located on microchromosomes, some showing association (Fig. 5a inset). R-banding pattern allowed the nine pairs of macrochromosomes to be precisely paired (Fig. 5b). In meiosis analysis, 9 macrobivalents and 12 microbivalents in diakinesis cells were observed (Fig. 6a). Heteromorphic sex chromosomes among the individuals were not detected.

***Cercosaura schreibersii albostrigatus* (2n = 44, 20M + 24m)**

Following the analysis of 21 Giemsa-stained metaphases, a karyotype consisting of 44 chromosomes was identified, comprising 20 macrochromosomes and 24 microchromosomes (Fig. 5c). All machrochromosomes are biarmed except pairs 9 and 10 that are acrocentrics. The microchromosome complement includes 12 pairs of acrocentrics. In diakinesis cells, 10 macrobivalents and 12 microbivalents were visualized (Fig. 6b). Heteromorphic sex chromosomes were not detected.

***Neusticurus bicarinatus* (2n = 44, 20M + 24m)**

The analysis of 10 conventionally stained metaphases revealed a karyotype consisting of 44 chromosomes, comprising 20 macrochromosomes and 24 microchromosomes (Fig. 5d). Between the biarmed macrochromosomes, the first pair is a large metacentric, the pairs 2 to 6 and 8 to 10 include submetacentrics and subtelocentrics, and, finally, the pair 7 is acrocentric. Ag-NORs were detected on the long arm of a biarmed macrochromosome pair (Fig. 5d inset). In meiosis, 10 macrobivalents and 12 microbivalents in diakinesis and 22 chromosomes in metaphases II cells were observed (Fig. 6c, d). Heteromorphic sex chromosomes were not detected.

***Placosoma cordylinum* (2n = 44, 20M + 24m)**

In a total of 20 Giemsa-stained metaphases we found a karyotype formed by 44 chromosomes, including 20 macrochromosomes and 24 microchromosomes, some clearly biarmed (e.g. pairs 1, 3, 4 and 11; Fig. 5e). Meiotic analyses revealed the presence of 10 macrobivalents and 12 microbivalents in diakinesis-stage cells (Fig. 6e). No heteromorphic sex chromosomes were identified.

***Placosoma glabellum* (2n = 58)**

Analysis of 31 conventionally stained metaphases revealed a karyotype comprising 58 chromosomes showing gradual decrease in size, with the majority being acrocentrics (Fig. 5f). Between the largest chromosomes, there is a subtelocentric pair that has clear short arms, classified as pair 1. The pair 2 is heteromorphic, because it is formed by a subtelocentric with short arms more evident than those of its acrocentric homologue, in both male and female cells. Among the microchromosomes, at least 4 pairs are biarmed. Meiotic spreads revealed 29 bivalents in diakinesis cells and 29 chromosomes in metaphase II, respectively (Fig. 6f, g). One of the largest pairs bears only homomorphic Ag-NORs (15 cells) on its short arms (Fig. 5f inset). Heteromorphic sex chromosomes were not observed.

### ﻿C-banding patterns

**Figure 7.**
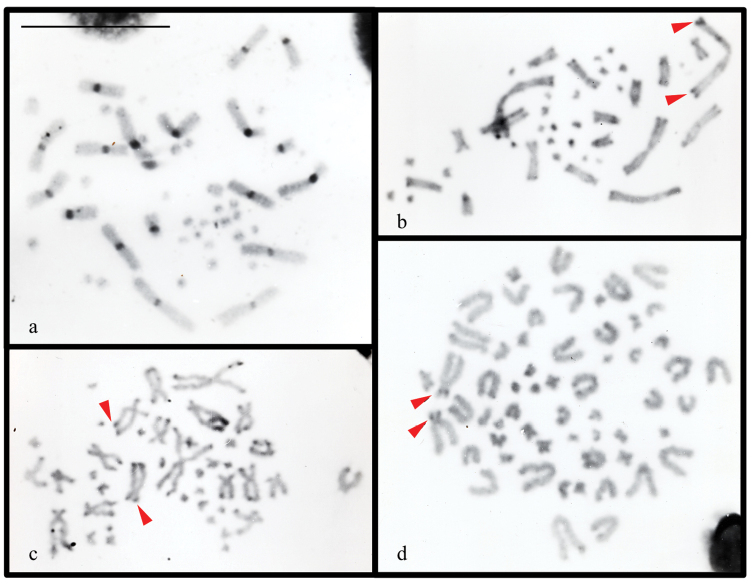
C-banding patterns in metaphases of **a.***Bachia bresslaui*; **b.***Cercosaura olivacea*; **c.***Neusticurus bicarinatus* and **d.***Placosoma glabellum*. The arrows point to lightly stained C-bands. Scale bar: 10 μm.

C-banding patterns were obtained for only four species analyzed in this study, revealing differences in both the amount and chromosomal distribution of constitutive heterochromatin. In *Bachia bresslaui*, conspicuous heterochromatic blocks were detected at pericentromeric regions of all macrochromosomes, with some microchromosomes also exhibiting faint signals in the same region (Fig. 7a). *Cercosaura olivacea* showed small C-bands in both the pericentromeric and telomeric regions of its macrochromosomes (Fig. 7b). In contrast, the constitutive heterochromatin in *Neusticurus bicarinatus* and *Placosoma glabellum* was predominantly distributed at telomeric regions (Fig. 7c, d, respectively). Specifically, *N. bicarinatus* displayed faintly stained C-blocks on the telomeric regions of the macrochromosomes, while *P. glabellum* exhibited lightly stained telomeric C-bands on most chromosomes, with the short arm of the subtelocentric pair 1 being a notable exception as it was almost completely heterochromatic (Fig. 7d).

## ﻿Discussion

Since the initial karyotypic description of a Gymnophthalmidae species (*Anadia bitaeniata* Boulenger, 1903) in the early 1970s, only 13 cytogenetic studies have been published to date (Table 1). Our study significantly expands the understanding of chromosomal diversity in these microteiid lizards by describing seven novel karyotypes for the subfamily Cercosaurinae. This contribution raises the total number of cytogenetically characterized species to 16, which still represents only about 8% of all species currently known in the subfamily.

It also provides new insights into chromosomal evolution, particularly within the Bachiini and Cercosaurini tribes. Cytogenetic studies have highlighted the high diversity and dynamic chromosome evolution within Gymnophthalmidae. Karyotypes exhibit a wide range of diploid numbers, from 2n = 34 (*Gymnophthalmus pleii*; Cole et al. 1990) to 2n = 62 (*Nothobachia ablephara*; Pellegrino et al. 1999a). While the presence of both macro- and microchromosomes appears to be a common feature across the family, the number, morphology, and relative sizes of these elements vary considerably among species (e.g. Pellegrino et al. 1999a, b; Yonenaga-Yassuda et al. 2005). A striking feature of Gymnophthalmidae, reinforced by our results, is the remarkable interspecific variation in diploid numbers, ranging in our sample from 2n = 32 in *Bachia dorbignyi* to 2n = 58 in *Placosoma glabellum*. Also, we found a wide range of variation within the same genera as *Bachia dorbignyi* (2n = 32, 18M + 24m) and *B. bresslaui* (2n = 46, 18M + 28m) and *Placosoma cordylinum* (2n = 44, 20M + 24m) and *P. glabellum* (2n = 58 with decreasing size chromosomes). Karyotypes with similar low diploid numbers such as that of *B. dorbignyi* were only recently found in the sister family Alopoglossidae, within *Alopoglossus angulatus* (Linnaeus, 1758) (2n = 24, 12M + 12m; Rachid et al. 2025), indeed representing the lowest 2n described for Gymnophthalmoidea to date.

In the latter case, Rachid et al. (2025) employed a probabilistic test to infer the ancestral diploid number for Gymnophthalmoidea, using chromosomal data available for 69 of its 502 species (representing approximately 13%). This approach revealed that the best-fitting model indicates an ancestral diploid chromosome number of 2n = 42 for the superfamily, implying that chromosomal fissions and fusions played a significant role in its karyotype evolution. However, the authors emphasize that this hypothesis requires confirmation by karyotyping additional species within the poorly characterized Gymnophthalmoidea.

We suggest the presence of a putative XX:XY sex mechanism in *Bachia dorbignyi* because we found a dot-like microchromosome exclusively in the two male specimens’ cells but not in those of female implying that it may represents the Y chromosome. Unfortunately, we were unable to obtain suitable meiotic spreads to identify a heteromorphic bivalent, as would be expected from the pairing of X and Y microchromosomes of markedly different sizes. However, this heteromorphism is not always detectable in all diakinesis or diplotene cells, likely due to the very small size of the microbivalent (see example in *Urostrophus vautieri* Duméril et Bibron, 1837, Pellegrino et al. 1999c; *Enyalius bilineatus* (Duméril et Bibron, 1837), Bertolotto et al. 2002). This sex chromosomal mechanism for *B. dorbignyi* is the first one with a dot-like Y described in Gymnophthalmidae. This sexual system is commonly found in other lizards’ families like those of Pleurodonta (e.g. Pennock et al. 1969; Bertolotto et al. 1996, 2002; Kasahara et al. 1996; Pellegrino et al. 1999c; Altmanová et al. 2016). Based on the known phylogenetic distribution of sex determination systems in squamates (Ezaz et al. 2009), the absence of heteromorphic sex chromosomes in the other six species of Cercosaurinae, as well as in other gymnophthalmids, may indicates that sex chromosomes are homomorphic as recently described in some species (e.g. *Scincus scincus* (Linnaeus, 1758) and *Tropidophorus baconi* Hikida, Riyanto et Ota, 2003; Kostmann et al. 2021). Further analyses using cytogenomics techniques may clarify the putative presence and nature of sex chromosomes in these species, and if the male heterogametic systems are homologous in all gymnophthalmids or evolved independently in different lineages. The presence of homomorphic sex chromosomes in the family would be interesting considering the lability of karyotype evolution and high rates of chromosomal rearrangements.

Ag-NORs may provide valuable information for karyotype characterization, particularly when distinct NOR-bearing chromosomes support phylogenetic relationships as seen in sister species of genera *Procellosaurinus* Rodrigues, 1991 and *Vanzosaura* Rodrigues, 1991 (Yonenaga-Yassuda et al. 1996), *Loxopholis* (Pellegrino et al. 1999b) and *Gymnophthalmus* (Yonenaga-Yassuda et al. 1999) in which Ag-NORs patterns revealed to be species-specific. Here, only in *Bachia bresslaui* and *Cercosaura olivacea*, we detected multiple Ag-NORs located at both macro- and microchromosomes which varied in number and position. *Bachia bresslaui* exhibited particularly high variability, showing intra- and interindividual polymorphism in the presence of active NORs in multiple chromosomal pairs. This variability, coupled with the detection of NORs on both homologues or in single chromosomes, may reflect a degree of genomic plasticity in the rDNA locus activation or positioning, potentially linked to differential gene expression (e.g. Kasahara et al. 1996; Yonenaga-Yassuda and Rodrigues 1999; Pellegrino et al. 1997, Rachid et al. 2025). Furthermore, this pattern of multiple Ag-NORs occurring on both macro- and microchromosome has also been found in others microteiids, such as *Nothobachia ablephara*, *Micrablepharus atticolus* Rodrigues, 1996, *Gymnophthalmus leucomystax* Vanzolini et Carvalho, 1991 and *Vanzosaura savanicola* Recoder, Werneck, Texeira, Colli, Sites et Rodrigues, 2014 (Pellegrino et al. 1999a; Yonenaga-Yassuda and Rodrigues 1999; Yonenaga-Yassuda et al. 1995, 1996, respectively).

In contrast, species such as *Neusticurus bicarinatus* and *Placosoma glabellum* exhibited a more conserved pattern, with a single pair of macrochromosome bearing the NORs. A similar pattern was also observed in other Cercosaurinae species, such as *Loxopholis osvaldoi* (Avila-Pires, 1995) (2n = 44, 20M + 24m; Pellegrino et al. 1999b) in which the NOR marker was located at the telomeres of the long arm of one of the largest pairs of submetacentric macrochromosomes. Furthermore, no cytogenetic variation was detected between the specimens of *Bachia bresslaui* and *Cercosaura olivacea*, despite their wide geographic distribution.

The heterochromatin patterns for the Gymnophthalmidae species *Bachia bresslaui*, *Cercosaura olivacea*, *Placosoma glabellum*, and *Neusticurus bicarinatus*, are herein described for the first time. The C-positive bands were predominantly located in the centromeric region in *B. bresslaui*, and at the telomeric regions in *C. olivacea*, *P. glabellum*, and *N. bicarinatus*. In *B. bresslaui*, the prominent centromeric C-bands observed in the macrochromosomes are otherwise known only in *Procellosaurinus*, a Gymnophthalminae distantly related genus (Yonenaga-Yassuda et al. 1996). Likewise, the faint C-banding patterns found in *C. olivacea*, *P. glabellum*, and *N. bicarinatus* are consistent with those reported for other gymnophthalmid species (e.g. Pellegrino et al. 1999a, b; Yonenaga-Yassuda et al. 2005), in which heterochromatin frequently serves structural or protective roles at chromosome´s telomeres (Janssen et al. 2018).

Despite the challenges in obtaining suitable material for cytogenetic analyses of these microteiids, we were able to describe RBG-banding patterns for *Cercosaura olivacea* only, pointing up the difficulty in obtaining high-quality metaphase spreads to be used in this technique (Yonenaga-Yassuda et al. 1988). RBG-banding enabled the accurate pairing of the macrochromosomes of *C. olivacea*, proving to be a useful cytogenetic tool (e.g. Pellegrino et al. 1999a, b; Yonenaga-Yassuda et al. 1999; Yonenaga-Yassuda et al. 2005).

From a taxonomic perspective, the data generated here highlight the potential utility of chromosomal features as diagnostic characters within Gymnophthalmidae as we detected cases of species-specific karyotypes. In particular, the distinct karyotype of *Bachia dorbignyi* (2n = 32) sets it apart from *Bachia bresslaui* (2n = 46) and reinforces the cytogenetic distinctiveness of these species and may aid in the delimitation of cryptic taxa. Similarly, chromosomal data for *Cercosaura schreibersii albostrigatus* (2n = 44) may contribute to the ongoing debate over the validity of subspecies within *Cercosaura schreibersii* (Torres-Carvajal et al. 2016), supporting future integrative taxonomic revisions.

## ﻿Conclusion

In summary, our study makes a pivotal contribution to the cytogenetic database of Gymnophthalmidae, a very diverse and exclusive family from the Neotropical region particularly for the Bachiini and Cercosaurini tribes, which had previously been underrepresented in the literature. Despite the difficulty in obtaining good quality chromosome preparations with a sufficient number of metaphases in these lizards, especially from intestinal epithelial cells, due to their very small body size (35 mm snout-vent length on average), the high degree of chromosomal diversity uncovered here corroborates the dynamic nature of genome evolution in this clade and opens new avenues for future research on speciation, sex determination, and genomic architecture in lizards. The incorporation of cytogenomic techniques and a broader taxonomic sampling will be essential to fully elucidate the evolutionary mechanisms shaping the karyotypes in this enigmatic group.

## ﻿Author contributions

LSFR, MTR, YYY, and KCMP conceived the study; LSFR and KCMP conducted the experiments; LSFR, YYY, and KCMP analyzed the data; MTR, YYY, KCMP provided the structure and resources; LSFR and KCMP wrote the manuscript. All authors read and approved the final version.

## Supplementary materials

Supplementary material 1 Species, specimen, sex and locality for the seven species of Cercosaurinae analyzed in this study Data type pdf Explanation note Species, specimen, sex and locality for the seven species of Cercosaurinae analyzed in this study. M = male; F = female; AM = state of Amazonas; BA = state of Bahia; GO = state of Goiás; MS = state of Mato Grosso do Sul; MT = state of Mato Grosso; RJ = state of Rio de Janeiro; SP = state of São Paulo; LG = Laboratory of Cytogenetics of Vertebrates (IBUSP, São Paulo Brazil); LGE = Laboratory of Evolutionary Genetics (UNIFESP, Diadema, Brazil); MTR = field number from Miguel Trefaut Rodrigues; CHTC = Coleção Herpetológica Teresa Cristina Sauer de Avila-Pires (UNIFESP); MZUSP = Museum of Zoology of the University of São Paulo. https://binary.pensoft.net/file/1508656This dataset is made available under the Open Database License (http://opendatacommons.org/licenses/odbl/1.0/). The Open Database License (ODbL) is a license agreement intended to allow users to freely share, modify, and use this Dataset while maintaining this same freedom for others, provided that the original source and author(s) are credited.Lucas S. F. Rachid, Yatiyo Yonenaga-Yassuda, Miguel Trefaut Rodrigues, Katia Cristina Machado Pellegrino
