# Supplementary material for: ﻿Seven new karyotypes for subfamily Cercosaurinae (Squamata, Gymnophthalmidae) with a synthesis of chromosomal data
Source: Comp Cytogenet. 2026 Jan 9;20:21–42. doi: 10.3897/compcytogen.20.170539 (PMC12811752; doi:10.3897/compcytogen.20.170539)
Supplement: Supplementary material 1 — Species, specimen, sex and locality for the seven species of Cercosaurinae analyzed in this study [file comparative_cytogenetics-20-021_article-170539__-s001.pdf]

Table S1. Species, specimen, sex and locality for the seven species of Cercosaurinae analyzed in this study. M = male; F = female; AM = state of Amazonas; BA = state of Bahia; GO = state of Goiás; MS = state of Mato Grosso do Sul; MT = state of Mato Grosso; RJ = state of Rio de Janeiro; SP = state of São Paulo; LG = Laboratory of Cytogenetics of Vertebrates (IBUSP, São Paulo Brazil); LGE = Laboratory of Evolutionary Genetics (UNIFESP, Diadema, Brazil); MTR = field number from Miguel Trefaut Rodrigues; CHTC = Coleção Herpetológica Teresa Cristina Sauer de Avila-Pires (UNIFESP); MZUSP = Museum of Zoology of the University of São Paulo.

Species	Specimen number	Sex	Locality
Bachia bresslaui	LG 655/MTR 916883/MZUSP 78211	M	Bataguassu/MS (−21.7180; −52.4225)
	LG 1167/MTR 968234	F	Caldas Novas/GO (−17.7333; −48.6166)
Bachia dorbignyi	LG 1193/MTR 977888/MZUSP 82647	M	Aripuanã/MT (−10.1666; −59.4500)
	LG 1354/MTR 977276/MZUSP 82422	F	Juruena/MT (−10.3236; −58.4927)
	LG 1355/MTR 977242/MZUSP 82421	M	
Cercosaura olivacea	LG 1038/MZUSP 79676	F	Fazenda Serrinha, Barão de Melgaço/MT (−15.8666; −55.7666)
	LG 1198/MTR 968153/ MZUSP 81553	M	Aripuanã/MT (−10.1666; −59.4500)
	LG 1091/MTR 7914/MZUSP 90020	M	Salvador/BA (−12.9666; −38.5000)
	LG 1142/MTR 7477/MZUSP 89955	M	Reservatório de Serra da Mesa, Niquelândia/GO (−14.4500; −48.4500)
	LG 1143/MTR 7433/MZUSP 90018	M	
Cercosaura schreibersii albostrigatus	LG 873/MTR 946138/MZUSP 78873	M	Alto Araguaia/MT (−17.3000; −53.2000)
Neusticurus bicarinatus	LG 1045/MTR 7476/MZUSP 89966	M	Manaus/AM (−2.9463; −59.9466)
Placosoma glabellum	LG 767/MTR 936149/MZUSP 78426	M	Ubatuba/SP (−23.3973; −44.9764)
	LG 768/MTR 936150/MZUSP 78427	F	
	LG 1131/MTR 7988/MZUSP 89980	F	Praia Vermelha do Sul, Ubatuba/SP (−23.5113; −45.1733)
	LG 776/MTR 946902/MZUSP 79751	F	Estação Ecológica da Juréia, Peruíbe/SP (−24.3335; −47.0084)
	LG 777/MTR 946143/MZUSP 78960	M	Praia de Boiçucanga, São Sebastião/SP (−23.7822; −45.6364)
	LG 940/MTR 946967/MZUSP 79752	M	Morro do Espia, Iguape/SP (-24.7000, -47.5666)
	LGE 89/MJS 798/CHTC 00844	F	Sítio Curucutu, São Bernardo do Campo/SP (−23.8942; −46.6176)
Placosoma cordylinum	LG 772/MTR 936146/MZUSP 78423	M	Teresópolis/RJ (−22.4000; −42.9500)
	LG 774/MTR 936145/MZUSP 78422	M	
	LG 954/MTR 7541/MZUSP 89962	M	
